# Evaluation of chemiluminescence, toluidine blue and histopathology for detection of high risk oral precancerous lesions: A cross-sectional study

**DOI:** 10.1186/1472-6890-12-6

**Published:** 2012-03-12

**Authors:** Shweta Ujaoney, Mukta B Motwani, Shirish Degwekar, Vijay Wadhwan, Prajakta Zade, Minal Chaudhary, Vinay Hazarey, Tushar P Thakre, Manju Mamtani

**Affiliations:** 1 Lata Medical Research Foundation, Nagpur, India; 2 Sharad Pawar Dental College & Hospital, Sawangi (Meghe), Wardha, India; 3 Government Dental College & Hospital, Nagpur, India; 4 12023 Waterway Rdg, San Antonio, TX 78249, USA

**Keywords:** Oral cancer, Leukoplakia, Screening, light-based methods

## Abstract

**Background:**

Early detection holds the key to an effective control of cancers in general and of oral cancers in particular. However, screening procedures for oral cancer are not straightforward due to procedural requirements as well as feasibility issues, especially in resource-limited countries.

**Methods:**

We conducted a cross-sectional study to compare the performance of chemiluminescence, toluidine blue and histopathology for detection of high-risk precancerous oral lesions. We evaluated 99 lesions from 55 patients who underwent chemiluminescence and toluidine blue tests along with biopsy and histopathological examination. We studied inter-as well as intra-rater agreement in the histopathological evaluation and then using latent class modeling, we estimated the operating characteristics of these tests in the absence of a reference standard test.

**Results:**

There was a weak inter-rater agreement (kappa < 0.15) as well as a weak intra-rater reproducibility (Pearson's r = 0.28, intra-class correlation rho = 0.03) in the histopathological evaluation of potentially high-risk precancerous lesions. When compared to histopathology, chemiluminescence and toluidine blue retention had a sensitivity of 1.00 and 0.59, respectively and a specificity of 0.01 and 0.79, respectively. However, latent class analysis indicated a low sensitivity (0.37) and high specificity (0.90) of histopathological evaluation. Toluidine blue had a near perfect high sensitivity and specificity for detection of high-risk lesions.

**Conclusion:**

In our study, there was variability in the histopathological evaluation of oral precancerous lesions. Our results indicate that toluidine blue retention test may be better suited than chemiluminescence to detect high-risk oral precancerous lesions in a high-prevalence and low-resource setting like India.

## Background

Oral malignancies continue to burden the clinical and economic dimensions of health care around the world [1,2]. In India, for example, oral cancers constitute 40% of all cancers and rank as the most common cancer in men and third most common cancer in women [3,4]. The reason why oral cavity cancers occupy a strategic position in the health care systems is that an early detection of these lesions is theoretically possible and practically useful [5-8]. Such early detection is generally associated with a high expectation of prevention of deformity, relapse and mortality [3,9].

Early detection of oral cavity carcinoma is, however, far from straightforward. Presence of precancerous lesions is not easy to detect due to a high likelihood of false-positivity. Histopathology continues to be used as the reference standard test [10]. However the difficulties in detecting early lesions with confidence [11] combined with the possible interrater variations of histopathological evaluations [12] compound the diagnostic challenges. For this reason, light-based methods [9,13,14] that visually highlight lesions are becoming popular as an adjunct for detection of precancerous lesions. Despite the expected theoretical benefit of these tests, Mehrotra et al [3] recently reported that these measures may not add a meaningful value to the simple diagnostic protocol of a detailed visual examination in a high prevalence setting. It has been argued [15,16] that the light-based methods are designed for screening rather than as a diagnostic aid in a tertiary care setting. However, in our experience and in conjunction with those reported by Mehrotra et al [3], these tests are currently used as diagnostic aids in tertiary care centers in India.

A possible explanation to the contested use of the light-based protocols for the diagnosis of precancerous lesions in high prevalence settings could be the variability in the histopathological evaluation. Current evaluation of the diagnostic/screening utility of these tests is contingent upon the assumption that histopathological evaluation is the reference standard. Arguably, however, if the histopathological evaluation is itself subject to errors then the estimates of the sensitivity and specificity of the light-based protocols can be expected to be biased. In this study, we considered the diagnostic performance of the light-based protocols without treating histopathological evaluation as a gold standard.

## Methods

### Study subjects

This study was conducted at the Oral Diagnosis, Medicine and Radiology Department of the Sharad Pawar Dental College, Sawangi, Maharashtra, India. Consecutive outpatients who visited the study center and who clinically presented with at least one precancerous lesion were recruited into this study. The exclusion criteria were: presence of frank malignancy (class I lesions based on Sciubba's [11] definition); known hypersensitivity to any ingredient or their analogues used during chemiluminescent light examination; any systemic disease that could obscure the true clinical presentation and interfere with or are contraindications to biopsy procedure; and any dental conditions such as orthodontic appliances or prostheses that may interfere with the examination.

### Study protocol

**Figure 1:**
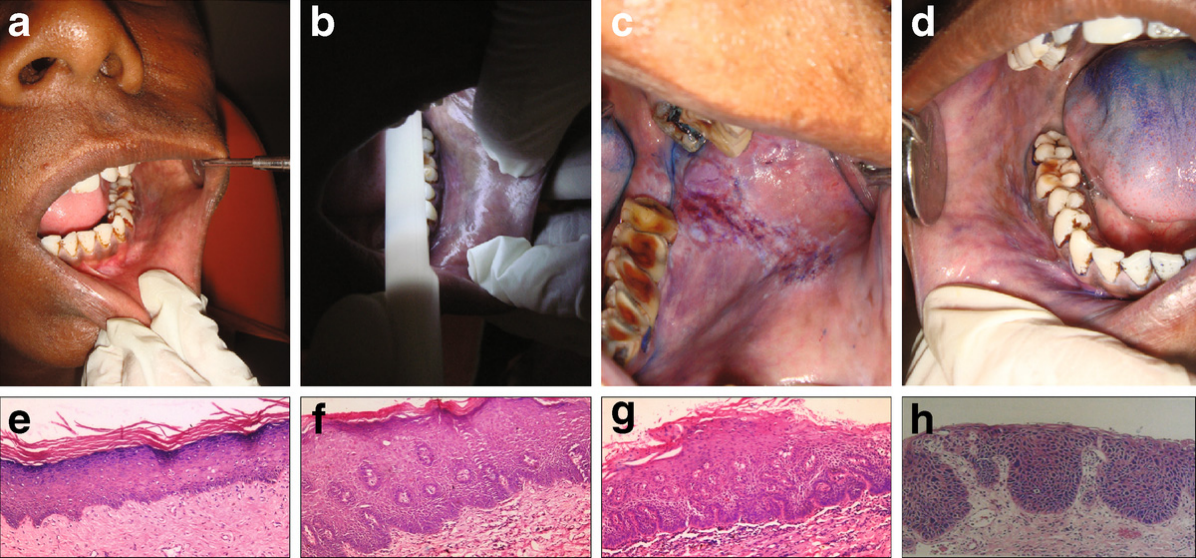
**Study protocol (A-D) and histopathological classification method (E-H)**. The study protocol included first **(A) **a regular visual inspection using standard operating procedures and regular light followed by examination using chemiluminescence **(B)**. This was followed by application of toluidine blue. The results of toluidine blue retention were seen as dark royal blue coloration **(C) **or faintly stained blue coloration **(D)**. Photomicrographs demonstrating the histopathological grading system which classified a lesion as 'no dysplasia' **(E) **if there was no cell atypia and no changes in architecture; 'mild dysplasia' **(F) **is there was keratosis, mild cellular atypia and architectural changes in the lower third of epithelium; 'moderate dysplasia' **(G) **if the architectural changes extended to the middle third of the epithelium; and 'severe dysplasia' **(H) **if there was a marked cellular atypia associated with architectural changes extending through the entire thickness of epithelium. All photomicrographs show hematoxilin and eosin staining and are depicted at 10× magnification.

A pre-enrolment screening questionnaire was used to record the history regarding the patients' complaints. After obtaining written informed consent the patients were enrolled in the study. The study was approved by the Ethical Committee of the Datta Meghe Institute of Medical Science, Wardha (Sawangi), India. Suspicious lesions were first identified with conventional visual examination under incandescent projected light and data including lesion characteristics like the location of the lesion, the type of lesion, the size, and the presence or absence of any adjacent satellite lesions were obtained. This was followed by an oral rinse with 1% acetic acid solution which was given to the patient to hold in the mouth for 30-60 seconds before expectorating. The oral cavity was then examined under conventional incandescent light for any new lesions that became visible or accentuated after the use of acetic acid (Figure 1A).

We then conducted two diagnostic tests and documented the results using one of the following three diagnostic protocols: chemiluminescent illumination system (CHEM, obtained from Vizilite^®^, Zila, Inc. Fort Collins, CO), toluidine blue retention test (TBLU) and a combination of chemiluminescence and toluidine blue retention test (CHTB, obtained from Vizilite PLus^®^, Zila, Inc. Fort Collins, CO). For CHEM protocol, we used The Vizilite^® ^light stick comprising an outer flexible capsule and a retractor (Figure 1B). Upon activation, the emanating light radiation (wavelength 430-580nm) was used to examine the oral cavity after dimming the room lights. The lesions that reflected the blue-white light were considered CHEM-positive. Any new lesion, not visible during conventional visual examination under incandescent light, but visible after chemiluminescent illumination test was noted and documented.

For TBLU protocol, the entire oral cavity was swabbed with 1% acetic acid solution and a pre-soaked swab of pharmaceutical grade toluidine blue was applied. Excess toluidine blue was removed using 1% acetic acid. Visual examination was then repeated under standard incandescent light to identify toluidine blue retention (Figure 1C) for each previously identified lesion and/or any new lesions subsequently found. Dark staining lesions were considered positive; faint lesions were considered equivocal; and those which did not take up the stain were considered negative. Using these categories, lesions were classified as TBLU-positive if it was observed to be positive and TBLU-negative if the result was either equivocal or negative. Finally, to classify using the CHTB protocol (Figure 1D), we considered a lesion to be CHTB-positive if it was both CHEM-positive and TBLU-positive; otherwise the lesion was considered to be CHTB-negative. Finally, incisional biopsy was performed on all lesions. All procedures were conducted during a single patient visit.

### Histopathological evaluation

Biopsy specimens were collected in 10% formalin solution and processed. Histopathologic evaluation was done by two senior Oral Pathologists blinded to the clinical findings. The first pathologist evaluated each specimen at two time points. The average interval between the two evaluations was 3 months. For all evaluations, the histopathologists used Smith and Pindborg's [17] scoring system which was based on 13 histopathological features. The total score ranged from 0 to 75 and, based on this total score, the histopathological grading was given as follows: no dysplasia (score 0-10, Figure 1E), mild dysplasia (score 11-25, Figure 1F), moderate dysplasia (score 26-45, Figure 1G) and severe dysplasia (score > 45, Figure 1H). We further reduced these evaluations to a binary classification scheme as high risk/low risk in accordance with the criteria set by the World Health Organization (WHO) classification [18].

### Statistical analyses

We studied the intra-and inter-rater agreement using Siegel and Castellan's fixed-marginal multi-rater kappa statistic, Bland-Altman plot and Pitman's variance ratio test for paired observations. The Siegel and Castellan's method of kappa estimation permits the estimation of per category kappa statistic (using the kap command in the Stata software package). To estimate the diagnostic performance of histopathological evaluations along with the three test protocols (CHEM, TBLU and CHTB) we did not make any *a priori *assumption about the reference standard. Such a representation of the data is amenable to latent class analysis (LCA) [19-22]. We used Hui and Walter's multinomial latent class model, the details of which are described elsewhere [23]. Briefly, if there are n dichotomous diagnostic tests, then there exist 2n + 1 unknown parameters to be estimated (n sensitivities, n specificities and prevalence) from a total of 2^n ^diagnostic combinations. The degrees of freedom for estimation of the parameters are, thus, 2^n^-1. Therefore this model can be used only if there are at least three tests (number of parameters to be estimated = 7 and degrees of freedom = 7). When the degrees of freedom exceed the number of parameters to be estimated the excess degrees of freedom can be used to test the goodness-of-fit of the latent class model. For latent class analyses, we used the latent1.exe program (Walter and Cook, personal communication). Other statistical analyses were conducted using the Stata 10.0 (Stata Corp, College Station, TX) statistical software package. Statistical significance was assessed at a type I error rate of 0.05.

## Results

We recruited 55 patients with 99 lesions. The characteristics of the study subjects and the lesions are described in Table 1. The majority of the study subjects were male and indulged in chronic tobacco use and/or betel nut chewing. There were ~70% subjects with two lesions. Also, 71% of the lesions involved the buccal mucosa (Table 1).

**Table 1:** Characteristics of the study subjects and samples

Characteristic	N^† ^or Mean*	%^† ^or SD*
Age (y)	44.4	17.1

Gender		
Males	51	92.7
Females	4	7.3

Personal habits		
Tobacco use		
Tobacco	16	29.1
Tobacco + lime	33	60.0
Snuff	2	3.6
Betel nut		
Smoking	35	63.6
Bidi	6	10.9
Cigarettes	2	03.6
Alcohol	11	20.0

Number of samples		
1	14	25.5
2	38	69.1
3	3	5.5

Location of lesion		
Tongue	5	5.1
Palate	1	1.1
Buccal mucosa	70	70.7
Buccal vestibule	10	10.1
Commensural Mucosa	7	7.1
Retromolar area	1	1.0
Labial vestibule	5	5.1

^†^, for categorical variables; *, for continuous variables; SD, standard deviation; y, years

### Variability in reference standard evaluation

**Figure 2:**
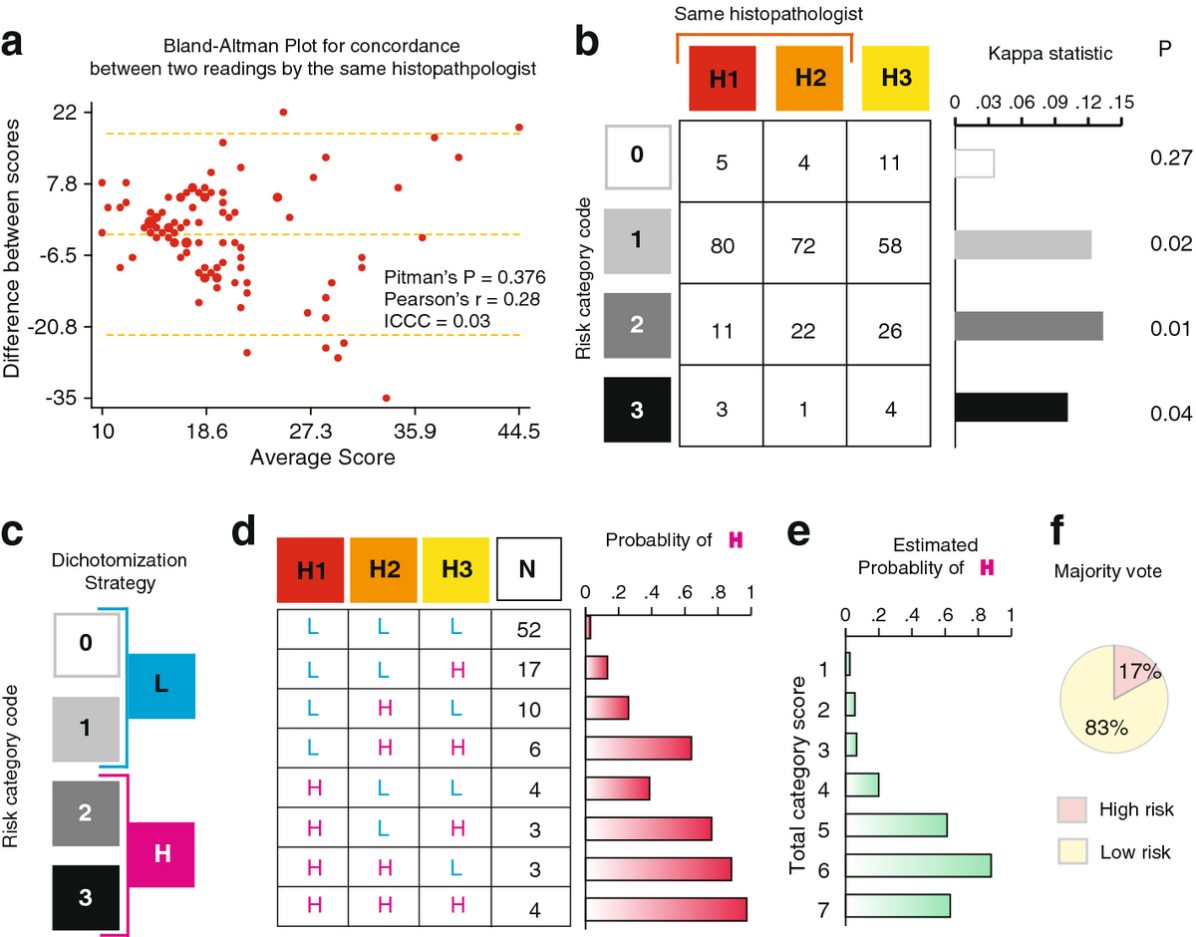
**Variability in the evaluation of precancerous lesions on histopathology**. **(A) **Intra-rater agreement on the Smith and Pindborg's scores at two time points spaced 3 months apart. The chart shows difference versus average Bland-Altman plot and the dotted lines represent the mean and 95% confidence interval for the difference. Pitman's P, significance value for difference in variability in two observations obtained using Pitman's variance ratio test. **(B) **Three histopathological evaluations (H1-H3) were available for each specimen. Two evaluations (H1 and H2) were contributed by the same pathologist at two different time points, while the third evaluation (H3) was contributed independently by a second histopathologist. Each lesion was classified as no (code 0), mild (code 1), moderate (code 2) or severe (code 3) dysplasia. These codes correspond to the Figure 1, panels E through H, respectively. Table shows the number of subjects classified based on each evaluation. Bar chart to the right shows the within-outcome category agreement using the kappa statistic. P, significance value of the kappa statistic. **(C) **Strategy used for dichotomization of the histopathology results. The colors match those in panel D. **(D) **Combinatorial representation of the three histopathological evaluations for latent class analyses. N, number of subjects. Bar chart to the right shows the estimated probability of a high-risk lesion (H) for given combination of the three histopathological evaluations. **(E) **Bar chart showing the association between total histopathological score and the estimated probability of a true high-risk lesion. No study subject had a score of 0, 8 or 9. **(F) **Pie chart showing the proportion of subjects classified as high risk (pink sector) or low risk (yellow sector) based on majority vote from the three histopathological evaluations.

We first considered if there existed intra-rater variability in the two histopathological evaluations by the same histopathologist. For this, we constructed a Bland-Altman plot on the paired observations provided by the same histopathologist (Figure 2A) and observed that there was neither a bias in the histopathologist's two evaluations nor a significant departure from variability at each time point as indicated by the Pitman's test. Despite this, however, the Pearson's correlation coefficient for scores at two time points by the same histopathologist was only 0.28 and the intraclass correlation coefficient was even lower (rho = 0.03, 95% confidence interval of rho 0.00-0.13). Together, these findings indicated that the two histopathological evaluations - even though from the same histopathologist -effectively behaved as statistically independent. Therefore, for the ensuing agreement analyses we treated these two evaluations and the evaluation by the other histopathologist as three independent evaluations.

The majority of the specimens were rated as mild by both the histopathologists (Figure 2B, code 1). We examined the inter-evaluation agreement for each category of the classification. In general, the kappa statistic was low (< 0.15) for all categories. However, the kappa statistic reached statistical significance for the mild, moderate, or severe categories (codes 1, 2, 3, respectively; Figure 2B). The overall agreement among the three evaluations was also low but statistically significant (kappa = 0.1126, *p *= 0.005). Together, these findings indicated a substantial intra-and inter-rater variability in the histopathological evaluations of the study specimens.

### Composite histopathological evaluation

Thus, we reasoned that the true histopathological evaluation for a given specimen would remain unknown. To use LCA, we needed to binarize the histopathological classification as shown in Figure 2C. Using this binarization scheme, we constructed eight combinatorial categories based on each histopathological evaluation (Figure 2D). The results of the LCA indicated that the estimated prevalence of the latent trait of a high-risk lesion was 20.8%. LCA predicted that the sensitivities of the three evaluations were 95.4%, 87.2%, and 71.4%, respectively, while the respective specificities were 50.4%, 63.1%, and 59.6%. Using these predictions, LCA estimated that the probability of a high-risk lesion was lowest when all the histopathological evaluations classified a specimen as a low-risk lesion, and highest when all the evaluations classified it as a high-risk lesion (bar graph in Figure 2D).

We then proceeded to evaluate the validity of a composite histopathological outcome. For this, we first generated the sum of codes ascribed to each specimen by all the three evaluations with the expectation that specimens with higher sums of scores (range 0-9) will have a higher likelihood of high-risk lesions. That indeed was the case (Figure 2E). One-way analysis of variance indicated that the total score explained 87.1% of the variability in the estimated probability of a high-risk lesion based on LCA. We then generated the majority vote from the three histopathological evaluations as follows: a lesion received as the histopathological majority vote (HPMV) the risk score seen in two or three evaluations. If all three evaluations yielded a different risk score for the same lesion then average risk score was taken as the HPMV. Using this composite measure, we observed that 17% specimens had a high-risk lesion (Figure 2F). This number corroborated the estimated prevalence of latent high-risk lesion trait using LCA.

### Comparison of diagnostic performance

We first compared the diagnostic performance of the three test protocols (CHEM, TBLU and CHTB) using the histopathological majority vote (HPMV) as the reference standard. We observed ( 2, column titled "Compared to HPMV") that CHEM had no specificity while the highest sensitivity and specificity was for TBLU and CHTB. We then conducted LCA for all the four dichotomous diagnostic test protocols. Considering the combinatorial results of the four test protocols together, we observed that (Table 2) the estimated prevalence of high-risk lesions in our study sample was 27.3% (95 CI 18.2%-36.3%). Interestingly, we observed that the histopathological majority vote had a high specificity but a low sensitivity. On the other hand, toluidine blue, alone or with chemiluminescence, had near-perfect sensitivity as well as specificity. Chemiluminescence alone had very low specificity. The overall goodness-of-fit of our LCA model was very good (χ^2 ^= 0.24, degrees of freedom = 6, *p *= 0.99).

**Table 2:** Diagnostic performance of the tests for high-risk lesions

Protocol	Parameter	Compared to HPMV	Using LCA
CHEM	Sensitivity	1.00 (0.82-1.00)	1.00 (0.99-1.00)

	Specificity	0.01 (0.00-0.06)	0.01 (0.00-0.04)

TBLU	Sensitivity	0.59 (0.36-0.78)	0.99 (0.92-1.00)

	Specificity	0.79 (0.69-0.87)	1.00 (0.99-1.00)

CHTB	Sensitivity	0.59 (0.36-0.78)	1.00 (0.99-1.00)

	Specificity	0.78 (0.68-0.86)	1.00 (0.99-1.00)

HPMV	Sensitivity	---	0.37 (0.19-0.55)

	Specificity	---	0.90 (0.83-0.97)

Prevalence of HRL		0.17 (0.10-0.24)	0.27 (0.18-0.36)

CHEM, chemiluminescence; TBLU, toluidine blue retention; CHTB, toluidine blue retention and chemiluminescence; HPMV, histopathology majority vote; LCA, latent class analysis; HRL, high risk lesion

## Discussion and Conclusions

We made three cardinal observations. First, for detection of precancerous lesions, there exists substantial intra-rater and inter-rater variation in the histopathological evaluation. Our results suggest that histopathology may be useful as a diagnostic test in demonstrably high degree of dysplasia or frank neoplasia but its value as a reference standard for diagnosis of low-risk precancerous lesions is questionable. Consequently, the use of histopathology as a reference standard against light-based assistance for diagnosis of high-risk lesions may lead to biased estimates of the diagnostic performance of these measures.

Second, we observed widely differing estimates of the sensitivity and specificity of the studied diagnostic protocols. However, caution needs to be exercised when reading and interpreting the results of latent class modeling [24-27]. A substantially different estimate of sensitivity (or specificity) for a test from that for the other tests can result from two scenarios: a) if the test is diagnostically inferior as compared to the rest; and b) if the test is using different criteria for classification of the disease state. In our case, the results do not necessarily imply that TBLU and CHTB are diagnostically superior to histopathology - rather it is possible that these tests use totally different criteria that do not compare with those used by histopathology. Nevertheless, our results clearly demonstrate (Table 2) that one of the main reasons for the controversial estimates of the diagnostic performance of light-based aids may be the classification method employed for the reference standard.

Third, a comparison of the diagnostic performance of TBLU and CHTB consistently indicated that use of CHEM may be somewhat redundant. From a primary health care perspective this finding is important since it will reduce the cost of diagnostic evaluation considerably by restricting the use of the more expensive component. Indeed the estimates of sensitivity and specificity of TBLU observed in this study are comparable with or better than those of other more expensive protocols like autofluorescence [28,29], photodynamic diagnosis [30], and chemiluminescence [31]. Our results are in agreement with the findings of Epstein et al which show that toluidine blue retention test holds promise as a screening tool for high-risk oral precancerous lesions since it can reduce a large number of unnecessary biopsies [32]. Concurring with other studies [33,34], our results encourage consideration of TBLU as a viable and feasible screening method in high-prevalence and low-resource scenarios like India.

There are important limitations of this study. First, as with the Mehrotra et al [3] study, our study recruited patients with a suspicion of a precancerous lesion for the reasons of feasibility as observed elsewhere [35]. However, the protocol did preclude visually negative patients that could have been later detected by at least one of the diagnostic methods. Our estimates of high sensitivity may also partially reflect this spectrum bias thereby limiting a ready generalization of the results. Second, the study sample had an *a priori *high likelihood of a precancerous lesion. Therefore our study design does not permit a full evaluation of the screening performance of these tests but rather considers them in the more practical scenario of a tertiary care setting as a diagnostic aid.

In summary, our findings support those of Mehrotra et al [3] and demonstrate that improvements are needed for histopathological evaluation of precancerous lesions - especially, low risk lesions. Our findings also suggest that toluidine blue retention may be considered as a diagnostic strategy for oral cancers in countries like India. More robust and larger studies are required to assertively and definitively answer questions related to the screening use of these tools in high prevalence settings.

## Competing interests

The authors declare that they have no competing interests.

## Authors' contributions

SU conceptualized the study, collected the data, conducted analyses and wrote manuscript. MBM conceptualized the study and reviewed manuscript. SD helped in conceptualizing the study, supported the administrative conduct of the study and reviewed the manuscript. VW and PZ conducted the histopathlogical assessments and reviewed the manuscript. MC contributed to the histopathological quality assessment and reviewed the manuscript. VH provided academic, administrative and conceptual support to the manuscript. TPT wrote and reviewed the manuscript. MM conducted the statistical analyses and prepared the first draft and subsequent revisions of the manuscript. All authors read and approved the final draft.

## Pre-publication history

The pre-publication history for this paper can be accessed here:

http://www.biomedcentral.com/1472-6890/12/6/prepub
